# Psychobiological Effects of Prenatal Glucocorticoid Exposure in 10-Year-Old-Children

**DOI:** 10.3389/fpsyt.2012.00104

**Published:** 2012-12-10

**Authors:** Katja Erni, Luljeta Shaqiri-Emini, Roberto La Marca, Roland Zimmermann, Ulrike Ehlert

**Affiliations:** ^1^Department of Clinical Psychology and Psychotherapy, Psychological Institute, University of ZurichZurich, Switzerland; ^2^Department of Obstetrics, University Hospital ZurichZurich, Switzerland

**Keywords:** prenatal glucocorticoids, acute psychosocial stress, psychobiological reactivity, HPA axis, children

## Abstract

**Background:** Prenatal stress seems to have long-lasting effects on biological and psychological processes of the offspring. However, to date, there have been no studies investigating the effects of prenatal glucocorticoid exposure on psychological, endocrine, and autonomic responses to a standardized psychosocial stress test in children. **Methods:** A sample of 115 healthy, 10-year-old children was examined. The Glucocorticoids + Tocolytics group was characterized by tocolytic treatment of the mothers due to preterm labor (*n* = 43). In addition, the pregnant women received glucocorticoid treatment in order to accelerate fetal lung maturation in case of preterm birth. The first comparison group (Tocolytics) consisted of children whose mothers also experienced preterm labor, but did not receive glucocorticoid treatment (*n* = 35). In the second comparison group (CONTROL), children whose mothers had a complication-free pregnancy were assessed (*n* = 37). Psychological parameters (stress appraisal and mood) using self-report questionnaires as well as salivary cortisol, salivary alpha-amylase, and heart rate were measured during a standardized psychosocial stress test (Trier Social Stress Test for Children). **Results:** Group comparisons revealed that a subscale of stress appraisal, control expectancies, significantly differed in children who were prenatally exposed to glucocorticoids as compared to both comparison groups (*F* = 4.889, *p* = 0.009). Furthermore, significant differences between the groups were revealed for salivary cortisol. With respect to overall stress appraisal and heart rate, trends toward significance were observed between the three groups. **Conclusion:** At the age of ten, those children who have been exposed to prenatal maternal glucocorticoids show changed psychobiological stress reactivity to a standardized psychosocial stress test as compared to control children.

## Introduction

Different lines of research in animals and humans indicate that adverse prenatal conditions such as malnutrition, stress, or anxiety experienced by pregnant mothers as well as prenatal glucocorticoid exposure seem to have long-lasting negative effects on postnatal biological and psychological processes in the offspring (Beydoun and Saftlas, 2008; Cottrell and Seckl, 2009). Animal and human studies have shown that prenatal stress alters baseline and stress reactivity of the hypothalamic-pituitary-adrenal (HPA) axis (Kofman, 2002; O’Connor et al., 2005; Weinstock, 2005; Van den Bergh et al., 2008). For example, prenatal glucocorticoid exposure in preterm babies may have an effect of the saliva cortisol stress response at 4 months (Glover et al., 2005).

Only a few studies have examined the effect of prenatal stress on the autonomic nervous system (ANS) in humans. Findings indicate that prenatally stressed neonates have a higher heart rate as compared to prenatally non-stressed controls (Davis et al., 2004; John et al., 2007).

These effects may result from adverse influences on the fetal physiological system. To explain the associations between prenatal environmental events, altered fetal growth, and development, and later pathophysiology the concept of early life physiological “programing” or “imprinting” has been advanced. Programing reflects the action of a factor during a sensitive developmental period or “window” to affect the development and organization of specific tissues that are concurrently vulnerable, producing effects that persist throughout life (Barker, 1998, 2002, 2007). It is well-established that exposure to prenatal stress has programing effects on the brain and the HPA axis of the fetus (Seckl and Meaney, 2004; Lupien et al., 2009). Excess glucocorticoid levels in early life can permanently alter tissue glucocorticoid signaling. These effects may have short-term adaptive benefits, e.g., for preventing tissue adversity, but otherwise increase the risk of later disease (Cottrell and Seckl, 2009). A variety of naturally occurring stressors provoke maternal hormonal HPA axis activation. However, intra- and inter-individual psychological and physiological reactions are only comparable to a limited extent. Therefore, it is important to apply standardized stressors.

Some pregnant women who have an imminent risk of a preterm delivery are administered synthetic glucocorticoids for medical reasons. In such cases, prenatally administered synthetic glucocorticoids accelerate fetal lung maturation, which is required for extra-uterine survival of preterm born babies (NIH Consensus Development Conference, 1995). Prenatal corticosteroid therapy is associated with a decrease in the incidence of respiratory distress syndrome (RDS), and therefore with better newborn survival (Liggins and Howie, 1972; Baud et al., 2000). Betamethasone belongs to the group of synthetic glucocorticoids that cross the placental barrier and stimulate the fetal HPA axis (Benediktsson et al., 1993). Therefore, the application of synthetic glucocorticoids (e.g., betamethasone) acts as a standardized pharmacological stressor and mimics the effects of a naturally occurring stressor. In this way it is possible to overcome the problems of subjectivity and diversity in prenatal stressors.

Despite the benefits of an increased survival rate in neonates, recent animal and human literature shows that betamethasone application during pregnancy provokes a variety of adverse physiological as well as psychological effects (e.g., Moss et al., 2005). Studies have shown effects of betamethasone on brain development in humans (Modi et al., 2001), cardiovascular risk factors (Dalziel et al., 2005) in humans, or increased postnatal symptoms of anxiety in mice (Rayburn et al., 1997). Moreover, after prenatal glucocorticoid administration aggressive/destructive, distractible, and hyperkinetic behavior at ages 3 and 6 years (French et al., 2004) have been found.

To our knowledge, there have been few studies examining the effects of prenatal glucocorticoids on stress reactivity in humans (e.g., Glover et al., 2005). Findings suggest that prenatal betamethasone treatment may lead to a suppression of HPA stress reactivity in healthy premature infants (Davis et al., 2004, 2006) as well as in healthy newborns born after 34 weeks of gestation (Schaeffer et al., 2009). A recent study found that full-term infants exposed to prenatal betamethasone showed a larger cortisol response to the heel stick procedure (Davis et al., 2011). To date, no study has examined the effects of prenatal stress, in particular prenatal glucocorticoids, on psychological parameters of a stress response in healthy subjects.

This is the first study to examine the long-lasting effects of prenatal glucocorticoids on psychobiological stress reactivity in response to a standardized psychosocial stress test in 10-year-old children. To this aim, we examined children whose mothers received glucocorticoid medication during the second trimester of pregnancy but delivered a healthy child after the 34th week of gestation. Glucocorticoid administration during pregnancy may mimic the consequences of serious stress on the unborn child. We hypothesized that children who were exposed to such prenatal stress would show altered psychological, endocrine, and autonomic responses to a standardized psychosocial stress test. We compared these children with a group of children whose mothers showed comparable medical complications during pregnancy but did not receive glucocorticoid treatment, as well as with a group of children whose mothers had complication-free pregnancies.

## Materials and Methods

### Participants

Recruitment was carried out with the help of medical staff, who screened medical records for 10-year-old children whose mothers showed preterm labor and received steroidal or non-steroidal treatment during pregnancy, or pregnancies without any complications. Additionally, subjects were recruited through posted announcements and flyers. The major criteria for eligibility were verified by a telephone screening. Subsequently, all medical records were screened to verify the criteria before the children and their mothers definitely were included in the study.

The final study sample consisted of 115 healthy children (the sample was part of a study with a total of 135 children). Eighty-three children were recruited by medical screening and 52 were recruited by posted announcements and flyers. We only included children and parents for statistical analyses who filled in all questionnaires and for whom analyses of saliva parameters were possible. All children showed comparable levels of development and education as measured by interviews. The Glucocorticoids + Tocolytics (GC + TOC) group (*n* = 43; 21 girls and 22 boys) was characterized by a standard tocolytic treatment (e.g., magnesium) of the children’s mothers due to signs of preterm labor during the second trimester of pregnancy. Commonly administered tocolytics were beta mimetics, magnesium sulfate, calcium channel blockers, and prostaglandin synthetase inhibitors (Zimmermann, 2006). In addition, the pregnant women received glucocorticoid treatment (betamethasone) in order to accelerate fetal lung maturation in case of preterm birth. The Tocolytics group consisted of 35 children (22 girls and 13 boys) whose mothers also had signs of preterm labor during the second trimester of pregnancy. These mothers, like the mothers of the GC + TOC group, were administered tocolytic treatment, but did not receive glucocorticoid treatment. In both of these groups, only pregnant women were included with no other reasons (e.g., premature rupture of membranes) for tocolytic treatment than preterm labor as reported in medical records. Administration of GCs was dependent on the decision of the treating physician. The GC + TOC and the TOC only group did not significantly differ in length of treatment with tocolytics. The CONTROL group consisted of 37 children (23 girls and 14 boys) whose mothers experienced a complication-free pregnancy. None of the children of the three groups developed complications after birth (e.g., oxygen administration because of RDS) or any other physical disease. Exclusion criteria for participation were maternal hypertension, diabetes mellitus as well as smoking during pregnancy, fetal structural anomaly, small for gestational age, birth before the 34th week of gestation, weight at birth below 2500 g, APGAR scores below 7, postnatal complications including administration of glucocorticoid medication, current intake of medication such as glucocorticoids or psychotropic drugs, and insufficient knowledge of the German language. All parents and children gave written informed consent. Parents were offered 50 Swiss Francs for travel cost compensation and children received a voucher for the amount of 60 Swiss Francs for their participation. The study was approved by the Ethics Committee of the Canton of Zurich, Switzerland and was conducted in accordance with the Declaration of Helsinki.

### Study protocol

Data assessment took place in the lab of the Institute of Psychology at the University of Zurich starting between 1:00 p.m. and 5:00 p.m. in order to control for diurnal variation in salivary cortisol (Kudielka et al., 2003). Children and parents received detailed information about the course of the study and gave written informed consent. During the first appointment, anamnestic data of parents and children were obtained. Moreover, data for the description of the socioeconomic status (SES) were collected, which was built from the school education of the mother and the professional position of the father (Largo et al., 1989). In addition, children’s general intelligence was measured. Upon arrival for the second appointment, children were fitted with an ambulatory electrocardiographic device, in the presence of the accompanying parent. Subsequently, the accompanying parent received detailed information about the methods of the acute psychosocial stress test and gave their written consent for the stress paradigm to be conducted with their children. Following an adaptation period, the children rated their mood with an adjective check list. Additionally, the first saliva sample, to assess endocrine stress reactivity, was obtained. Immediately afterward, children were introduced to the psychosocial stress test. At the end of the 5 min preparation phase, immediately before retelling the story, all subjects completed a self-report transactional stress questionnaire and a mood adjective list. Then, the second saliva sample was obtained. After the psychosocial stress test, another saliva sample was collected and the children filled in the adjective check list once more. Further saliva samples were obtained within 1 h after the end of the psychosocial stress test. At this time, the children took the ambulatory electrocardiographic device off.

### Psychosocial stress test

To induce acute psychosocial stress, we used the “Trier Social Stress Test for Children” (TSST-C; Buske-Kirschbaum et al., 1997), which is an adapted version of the well-established “TSST” (Kirschbaum et al., 1993). The TSST-C consists of a short introduction (Intro), which is followed by a 4 min preparation phase, 5 min to finish a story in a free speech, and a 5 min mental arithmetic task in front of an audience. At the beginning, the children were brought into a room where two individuals, the so-called committee, were sitting at a desk. During the Intro, the children listened to the beginning of a story and were asked to finish the story in front of the committee and to make the ending as exciting as possible. The children were told to try to outperform other participants of the study. Additionally, they were informed that their story-telling performance would be videotaped for further analysis of their behavior. After the 4 min of preparation time, the children were exposed to the 5 min story-telling task. Whenever a child finished the story within less than 5 min, he/she was asked to continue. After 5 min, the committee asked the children to count down from 758 in increments of 7 in a fast and accurate manner. Whenever they made a mistake, they had to start again at 758. At the end of the examination, the participants were debriefed, first with the children alone and then together with the accompanying parent. The committee explained the objective and methods of the TSST-C. They told each child that he/she had done a very good job and gave them sweets.

### Psychological measures

Stress appraisal was assessed using the Primary Appraisal and Secondary Appraisal Scale (PASA; Gaab et al., 2005). This questionnaire is based on the transactional stress model (Lazarus and Folkman, 1984) and assesses relevant cognitive processes during the anticipation of a stressful situation. The PASA consists of 16 situation-specific items forming four primary subscales; challenge and threat (primary appraisal), self-concept of own competence and control expectancy (secondary appraisal). The two secondary scales, primary and secondary appraisal, can be summarized to form an overall stress appraisal. The ability of children in this age group to understand these questions was checked in a pilot study (data not presented). Children’s mood was assessed using a multidimensional adjective check list “Eigenschaftswörterliste” (EWL-KJ; Janke and Janke, 2005). The EWL-KJ is comprised of 40 adjectives, which are grouped into the following 10 subscales: recreation, good mood, activity, excitement, bad mood, anger, aggression, anxiety, depression, deactivation. These subscales can be combined to yield two global scales, which are termed positive emotionality and negative emotionality. The responses describe the children’s emotion at that moment. Intelligence level was measured with the Raven’s Colored Progressive Matrices (CPM; Raven et al., 1998). CPM is an instrument to assess non-verbal intelligence and logical reasoning of children from 3.9 years up to the age of 11.8 years. This test consists of 36 items in 3 sets with 12 items per set. A single raw score is computed of all correct items and that can be converted to a percentile based on normative data collected from various groups.

### Physiological measures

The children were asked to abstain from physical exercise (48 h), caffeinated beverage, black tea, and chewing gum (18 h), as well as brushing their teeth and food intake (1 h) prior to the experiment. A total of nine saliva samples for the assessment of cortisol and alpha-amylase levels were collected: 1 min before subjects were presented with the Intro in order to assess baseline levels, just before the TSST-C and immediately thereafter, as well as 5, 10, 20, 30, 45, and 60 min after completion of the TSST-C. Saliva was collected using Salivette collection devices (Sarstedt, Sevelen, Schweiz) and was stored at −20°C. Saliva samples were thawed out and spun at 3000 rpm for 5 or 10 min in order to obtain low-viscosity saliva. Salivary free cortisol was analyzed by using a commercially available competitive chemiluminescence immunoassay (LIA; IBL Hamburg, Germany). Intraassay and interassay coefficients of variation were below 10% (Dressendorfer et al., 1992; Nater et al., 2006). For the determination of alpha-amylase activity, an assay was used, which has been described previously (Nater et al., 2006). In brief, salivary alpha-amylase was tested with a kinetic-colorimetric test using an assay-kit (Roche, Rotkreuz, Switzerland) and then read in an ELISA-reader-apparatus (Synergy-HT, Biotec. ORT). Electrocardiographic data were recorded with the LifeShirt system 200 (Vivometrics, Ventura, CA, USA). After data editing, heart rate was determined for 1 min intervals. For statistical analysis, heart rate was averaged for the 5 min immediately before the TSST-C, during the 10 min of the TSST-C – five minutes story and five minutes arithmetic task –, and five minutes thereafter.

### Statistical analyses

Data were analyzed using SPSS statistical software package version 17.0 (Chicago, IL, USA). Normal distribution of the data was verified by the Kolmogorov–Smirnov test and homogeneity of variance by the Levine’s test before the statistical procedures were applied. Analyses of variance for repeated measures were computed in order to investigate whether the stressor TSST-C provoked a significant change in the psychobiological stress response parameters across the various time points. All statistical analyses were performed controlling for covariates (weeks of gestation, weight at birth, length at birth, whether the parents were living together at time of the examination and current physical and psychological symptoms of parents as assessed using the Brief Symptom Inventory (BSI; Table 1; Franke, 2000)). If the assumption of sphericity was violated (Huynh–Feldt), all reported results were corrected by the Greenhouse–Geisser procedure. *Post hoc* analyses using LSD were conducted. For endocrine variables the areas under the response curve were calculated with respect to ground (AUCg) and to increase (AUCi) using the trapezoidal method as an indicator of the physiological stress response (Pruessner et al., 2003). AUC measures were computed between rest (1 min before the intro) and 60 min after stress (60 min after completion of the TSST-C; AUCg), between rest (1 min before the intro) and immediately post-stress (1 min after completion of the TSST-C; AUCi increase). These often described statistical methods were used to characterize the total amount of cortisol secretion, the increase of cortisol secretion, and the course of the cortisol levels over time (see Campbell and Ehlert, 2012). All results are presented as means and standard errors of means (SEM) unless otherwise indicated. All testing was two-tailed with the significance level set at *p* ≤ 0.05.

**Table 1:** **Sample characteristics of children with glucocorticoid and tocolytic administration (*n* = 43), children in the tocolytics group (*n* = 35), and children in the CONTROL group (*n* = 37; means ± SEM)**.

	GC + TOC group (*n* = 43)	TOC group (*n* = 35)	CONTROL group (*n* = 37)	*p*
Weeks of gestation (in days)	263.927 (±2.191)	265.263 (±2.123)	277.081 (±1.897)	***
Weight at birth (in grams)	3118.658 (±83.023)	3006.528 (±82.295)	3410.333 (±74.676)	**
Length at birth (in centimeters)	47.878 (±0.3892)	47.986 (±0.429)	49.754 (±0.324)	***
APGAR 1, 2, 3	8.025 (±0.150)	8.342 (±0.127)	8.444 (±0.122)	ns.
	9.150 (±0.105)	9.3156 (±0.093)	9.417 (±0.108)	ns.
	9.500 (±0.095)	9.553 (±0.098)	9.778 (±0.0703)	ns.
Age at assessment, (in months)	125.020 (±0.627)	125.05 (±0.630)	125.680 (±0.604)	ns.
Sex (girls/boys)	21/22	22/13	23/14	ns.
Body mass index (in kg/m^2^)	16.698 (±0.346)	16.486 (±0.367)	17.150 (±0.276)	ns.
Intelligence score (CPM)	103.670 (±2.802)	103.180 (±2.702)	100.190 (±2.735)	ns.
Socioeconomic status (SES) of parents	4.950 (±0.264)	5.050 (±0.291)	5.160 (±0.188)	ns.
Parents were living together or not	39/4	22/13	32/5	*
Brief symptom inventory (BSI) mothers	3.822 (±0.574)	3.255 (±0.617)	2.384 (±0.407)	ns
Brief symptom inventory (BSI) fathers	2.799 (±0.518)	2.453 (±0.491)	1.714 (±0.202)	ns

*n* = valid cases, SEM = standard error of means, ***p < 0.001, **p < 0.01, *p < 0.05.

## Results

### Sample characteristics

A total sample of 115 children was analyzed. The three groups did not differ significantly from each other with respect to sample characteristics (Table 1) and sex (*p* = 0.362). Significant differences between the groups were found for weeks of gestation, weight at birth, length at birth, and for whether or not parents were living together. There were no significant differences between the GC + TOC group and the TOC group regarding weight at birth and length at birth.

### Psychological stress responses

#### Primary appraisal and secondary appraisal scale

**Figure 1:**
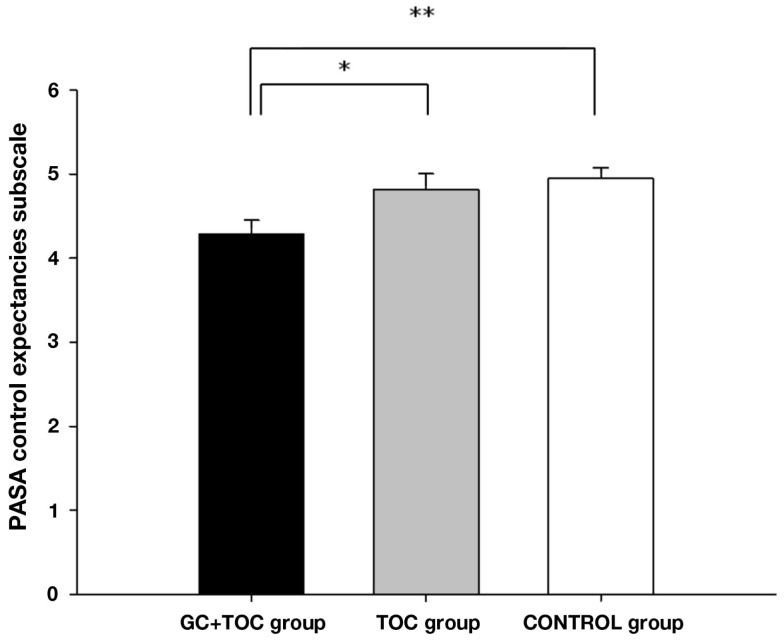
PASA control expectancies subscale before the TSST-C (MEANS ± SEM) in children with glucocorticoid administration and tocolytic (GC + TOC; *n* = 43), in children of the TOC group (*n* = 35), and in children of the CONTROL group (*n* = 37) (**p < 0.01, *p < 0.05).

The ANOVA for comparing control expectancies between the groups revealed significant differences [*F*(2, 112) = 4.889, *p* = 0.009, partial η^2^ = 0.080], with the GC + TOC group exhibiting the lowest control expectancies as compared to the TOC group and the CONTROL group (*post hoc* analysis, *p* = 0.023 and *p* = 0.004, respectively; Figure 1). A trend toward significance in the overall stress appraisal was observed [*F*(2, 112) = 2.354, *p* = 0.100, partial η^2^ = 0.040]. The GC + TOC group showed the highest stress index (Table 2).

**Table 2:** **Anticipatory stress appraisal (PASA) before the TSST-C and mood of children (EWL-KJ) at baseline, during and after the TSST-C in children with glucocorticoid (GC) and tocolytic (TOC) administration (*n* = 43), children of the TOC group (*n* = 35), and children of the CONTROL group (*n* = 37; means ± SEM)**.

	GC + TOC group (*n* = 43)	TOC group (*n* = 35)	CONTROL group (*n* = 37)	*p*
PASA “control expectancies”^a^	4.280 (±0.170)	4.810 (±0.190)	4.950 (±0.120)	*
PASA “stress appraisal”^b^	−0.830 (±0.470)	−2.38 (±0.520)	−1.420 (±0.530)	0.100
EWL-KJ “recreation”^a^
Pre	6.977 (±0.449)	7.053 (±0.364)	8.622 (±0.392)	*
Peri	4.116 (±0.487)	5.0571 (±0.438)	4.487 (±0.503)	
Post	5.558 (±0.502)	5.429 (±0.482)	6.297 (±0.515)	
EWL-KJ “anxiety”^a^
Pre	0.8370 (±0.191)	0.6316 (±0.175)	0.2162 (±0.088)	*
Peri	3.302 (±0.486)	2.0571 (±0.418)	4.1351 (±0.571)	
Post	1.419 (±0.328)	1.1143 (±0.301)	1.3784 (±0.350)	
EWL-KJ “global scale positive emotionality”^a^
Pre	24.535 (±0.969)	23.395 (±1.000)	26.676 (±958)	*
Peri	16.628 (±1.281)	19.229 (±1.218)	17.811 (±1.414)	
Post	19.837 (±1.179)	19.571 (±1.521)	20.460 (±1.326)	

*n* = valid cases; SEM, standard error of means; *p < 0.05; PASA, Primary Appraisal and Secondary Appraisal Scale; EWL-KJ, Eigenschaftswörterliste für Kinder und Jugendliche.

*^a^Higher scores indicate higher control expectancies, more recreation, higher anxiety, or higher emotionality*.

*^b^More negative scores indicate less stress appraisal*.

#### EWL-KJ

The TSST-C caused significant changes on all subscales as well as on the two global scales of the mood rating scale EWL-KJ (main effect of time; *p* < 0.010), with the exception of the subscale aggression (trend; *p* < 0.063). On the subscale recreation, the three groups differed significantly [*F*(4.000, 224.000) = 2.977, *p* = 0.020, partial η^2^ = 0.050]. The GC + TOC group was less relaxed compared to the TOC group and the CONTROL group. Significant differences between the groups were found for the global scale positive emotionality [*F*(4.000, 224.00) = 2.720, *p* = 0.031, partial η^2^ = 0.046). The GC + TOC group had less positive emotionality compared to the TOC group and the CONTROL group. On all other scales, no significant differences between groups were found (Table 2).

### Physiological stress responses

The participation in the TSST-C caused significant increases in all assessed physiological parameters (cortisol, alpha-amylase, heart rate; main effect of time; *p* < 0.001) in all three groups. No significant differences were observed among groups in cortisol or alpha-amylase levels and heart rate baseline levels before the TSST-C.

#### Cortisol

**Figure 2:**
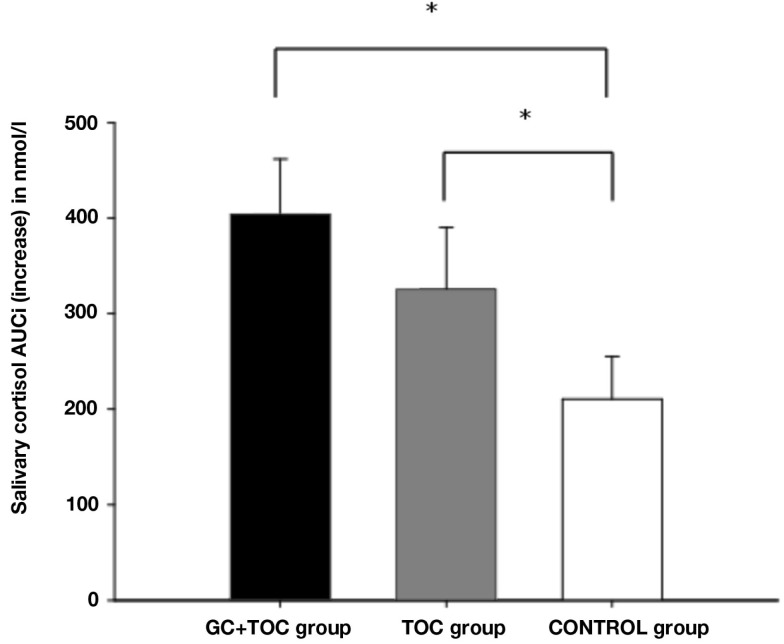
Salivary cortisol AUCi (increase) in response to the TSST-C (MEANS ± SEM) in children with glucocorticoid and tocolytic administration (GC + TOC; *n* = 43), in children of the TOC group (*n* = 35), and in children of the CONTROL group (*n* = 37) (*p < 0.05).

Results obtained from repeated measures ANOVAs indicated that the differences in salivary cortisol increase between the three groups revealed a trend toward significance [*F*(2, 112) = 2.804, *p* = 0.065, partial η^2^ = 0.048; Table 3]. The GC + TOC group showed significantly higher cortisol levels as compared to the CONTROL group (*post hoc* analysis, *p* = 0.021). Significant differences between the groups were obtained for both the total amount of cortisol [AUCg, *F*(2, 102) = 3.250, *p* = 0.043, partial η^2^ = 0.060] and the total amount of cortisol increase [AUCi, *F*(2, 102) = 3.225, *p* = 0.044, partial η^2^ = 0.059; Figure 2]. The total amount of cortisol was significantly higher in the GC + TOC group as compared to the CONTROL group (*post hoc* analysis, *p* = 0.025) as well as in the TOC group compared to the CONTROL group (*post hoc* analysis, *p* = 0.037). The GC + TOC group demonstrated a significantly steeper increase in cortisol than the CONTROL group (*post hoc* analysis, *p* = 0.021), and the TOC group showed a significantly steeper increase in cortisol compared to the CONTROL group (*post hoc* analysis, *p* = 0.048).

**Table 3:** **Measurement points (time in minutes) of the nine saliva samples and the assessed levels of cortisol in children with glucocorticoid (GC) and tocolytic (TOC) administration (*n* = 43), children of the TOC group (*n* = 35), and children of the CONTROL group (*n* = 37; means ± SEM)**.

Time in min	GC + TOC group (*n* = 43)	TOC group (*n* = 35)	CONTROL group (*n* = 37)	*p*
−20 (before Intro)	2.4493 (±0.17432)	2.6245 (±0.26633)	2.4727 (±0.25781)	ns.
−1 (before TSST-C)	3.8153 (±0.36500)	3.7169 (±0.43458)	2.9273 (±0.28680)	ns.
1	7.0298 (±0.62373)	7.6677 (±1.20104)	5.4535 (±0.57685)	ns.
5	9.5549 (±0.81413)	8.9511 (±1.28891)	6.4103 (±0.65242)	*
10	11.4595 (±0.99545)	9.2377 (±1.26703)	7.9470 (±0.93733)	ns.
20	11.3977 (±1.32406)	9.9309 (±1.56930)	7.5949 (±0.98617)	ns.
30	9.4193 (±1.37103)	8.0017 (±1.31556)	5.5414 (±0.66949)	ns.
45	6.2340 (±0.94999)	5.3543 (±0.72477)	4.1665 (±0.38167)	ns.
60	4.6002 (±0.53684)	4.1509 (±0.50658)	3.3966 (±0.26129)	ns.
AUCg	612.2349 (±62.1180)	548.4666 (±71.9300)	420.8790 (±38.5441)	*
AUCi	404.0442 (±57.7794)	325.3841 (±64.9740)	210.0442 (±57.7794)	*

*n*, valid cases; SEM, standard error of means; *p < 0.05.

#### Alpha-amylase

Repeated measures ANOVAs revealed no group differences in response to the TSST-C for salivary alpha-amylase [*F*(5.566, 311.710) = 1.080, *p* = 0.373]. Moreover, no significant differences between the three groups were obtained for the total amount of alpha-amylase (AUCg) and increase [AUCi; *F*(2, 102) = 0.602, *p* = 0.550, respectively *F*(2, 102) = 1.456, *p* = 0.238].

#### Heart rate

**Figure 3:**
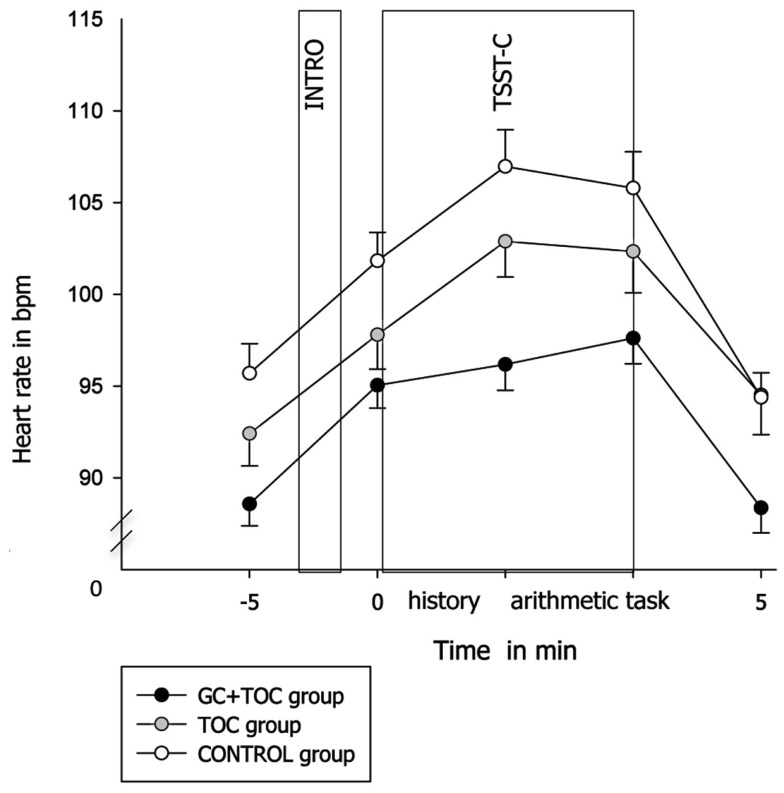
**Heart rate in response to the TSST-C (MEANS ± SEM) in children with glucocorticoid and tocolytic (GC + TOC) administration (*n* = 43), in children of the TOC group (*n* = 35), and in children of the CONTROL group (*n* = 37)**.

An ANOVA with repeated measures revealed a trend toward significance for heart rate increase in response to the TSST-C between the three groups [*F*(13.791, 861.947) = 1.615, *p* = 0.071, partial η^2^ = 0.025; Figure 3]. Heart rate was lower in the GC + TOC group than in the TOC group (*post hoc* analysis, *p* = 0.019) and CONTROL group (*post hoc* analysis, *p* < 0.001). Significant differences between the groups were obtained for both the total amount of heart rate [AUCg, *F*(2, 97) = 6.886, *p* = 0.002, partial η^2^ = 0.124], but not for the total amount of heart rate increase [AUCi, *F*(2, 97) = 0.604, *p* = 0.549]. The total amount of heart rate was significantly higher in the GC + TOC group as compared to the TOC group (*post hoc* analysis, *p* = 0.024) as well as compared to the CONTROL group (*post hoc* analysis, *p* = < 0.001).

## Discussion

To our knowledge, this is the first study to investigate the effects of prenatal glucocorticoids on psychological, endocrine, and autonomic responses to a standardized psychosocial stress test. The TSST-C induced significant changes in both psychological and biological parameters in all three groups. Children who were prenatally exposed to glucocorticoids showed lower control expectancies and higher stress appraisal. Moreover, these children showed higher salivary cortisol levels and lower heart rates than the children of the comparison groups. Not only children who were exposed to glucocorticoids during pregnancy but also children whose mothers received non-steroidal tocolytics showed a different cortisol stress reactivity compared to children from healthy pregnancies.

To the best of our knowledge, to date, no human studies have examined the association between prenatal glucocorticoid exposure and psychological stress response. The children who were prenatally exposed to glucocorticoids experienced the highest stress appraisal in comparison to the two comparison groups. They showed a lower appraisal of self-concept of own competence and control expectancy (secondary stress appraisal) in view of the stressful situation than of challenge and threat (primary stress appraisal). This mismatch results in a higher stress appraisal. Animal studies revealed that prenatal overexposure to glucocorticoids caused reduced coping under the condition of stress (Drake et al., 2007). Moreover, young adults whose mothers experienced psychosocial stress during pregnancy showed significantly higher levels of tense arousal (more jittery, less relaxed) during the TSST as compared to a control group (Entringer et al., 2009).

Our findings that the GC + TOC group showed a larger psychological stress response provides compelling evidence that this is an effect of the prenatal glucocorticoid exposure. The GC + TOC group differed in the psychological stress response as compared to the comparison group as well as compared to the TOC group. This is in line with other studies that found an association between prenatal stress and effects on psychological variables later in life. For example, a study found that prenatal exposure to elevated maternal cortisol, depression, perceived stress, and pregnancy-specific anxiety was associated with increased anxiety in children at 6–9 years of age (Davis and Sandman, 2012). Moreover, the findings of another study provide support that higher maternal pregnancy-specific anxiety is associated with increased negative temperament in the children (Blair et al., 2011).

The few published human studies on the effect of prenatal glucocorticoid exposure propose that this exposure may affect the HPA axis stress response in later life. For example, premature infants were found to exhibit a blunted reactivity of salivary cortisol in response to a pain-induced stress event (a heel stick blood draw) several days after birth (Davis et al., 2004). Similar results were found in neonates (Schaeffer et al., 2009). Findings therefore seem to suggest a suppression of the HPA axis stress reactivity in children who were prenatally exposed to glucocorticoids. These children seemed less able to regulate the stress response. Our findings, by contrast, suggest a hyper-responsiveness of the HPA axis in response to a standardized stress test. This is in line with a study where full-term infants exposed to prenatal betamethasone showed a larger cortisol response to the heel stick procedure as compared with a comparison group (Davis et al., 2011). Moreover, similar results were found in studies that examined the effects of prenatal stress on HPA stress reactivity, but define prenatal stress in a different way. In these studies, in comparison to controls, a higher cortisol reaction to vaccination was found in children whose mothers experienced high psychosocial stress during pregnancy (Gutteling et al., 2004) and a higher cortisol reaction to the TSST was found in young adults who experienced prenatal psychosocial stress (Entringer et al., 2009). Further evidence of an HPA axis dysregulation can be found in studies on the diurnal course of cortisol with prenatally stressed neonates and children (Lundy et al., 1999; Gutteling et al., 2005; Field et al., 2006) as compared to controls. The differences in hyper- vs. hypo-responsiveness of the HPA axis following stress may be the result of specific developmental processes. For example, it could be demonstrated in sheep at different age of assessment that HPA function undergoes developmental changes that are influenced by prenatal glucocorticoid exposure (Sloboda et al., 2002, 2007).

The children of the three groups showed different salivary cortisol patterns. We found significant differences in the salivary cortisol reactivity not only between the GC + TOC group and the CONTROL group, but also between the TOC group and the CONTROL group. It seems that the experiences of preterm labor acted as a stressor for the pregnant women and therefore acted as prenatal stress on the unborn child that had an influence on the salivary cortisol reactivity later in life.

As yet, the effect of prenatal glucocorticoid exposure on salivary alpha-amylase has not been investigated. A small number of studies showed that alpha-amylase increased in reaction to the TSST-C in children and adolescents (Gordis et al., 2006; Stroud et al., 2009; Strahler et al., 2010; Yim et al., 2010a,b), as we were also able to demonstrate in our study. A higher heart rate was observed in reaction to a heel stick blood draw in infants who received prenatal glucocorticoids as compared to controls (Davis et al., 2004). This is in line with another study (John et al., 2007) who found that neonates who were prenatally exposed to cocaine had a significantly higher heart rate following orthostatic stress than neonates without such a history. In contrast, no differences were found in the heart rate stress response to a psychological stressor in adults who were prenatally exposed to the Dutch famine (Painter et al., 2006).

To conclude, there is evidence that prenatal stress (e.g., prenatal glucocorticoid exposure or preterm labor) may be associated with a dysregulation of the HPA axis in the offspring. Furthermore, prenatal stress (e.g., prenatal glucocorticoid exposure) may be associated with a dysregulation of the autonomic reactivity in the offspring. Our findings could be framed in the fetal programing hypothesis of Barker (1998). It states that the environment *in utero* can alter the development of the fetus during particular sensitive periods, with a permanent effect on the organism in later years. Further studies are needed to examine the direction of the endocrine and autonomic dysregulations (i.e., hypo- or hyper-reactivity) after a stress provocation, in particular with respect to different age groups and definitions of prenatal stress.

The present study has several strengths. First, we used an objective standardized pharmacological stressor so as to overcome the problems of subjectivity and diversity in prenatal stressors. Second, we included three groups of children in order to compare two different definitions of prenatal stress, namely preterm labor and glucocorticoid administration. Moreover, a comparison group was added with children whose mothers did not have any pregnancy complications. This allows more possibilities for interpreting and comparing the findings. Third, variables that may confound the examined variables were excluded or controlled, e.g., weight at birth below 2500 g, RDS after birth or weeks of gestation, as well as weight and length at birth. Fourth, we examined the psychological and biological stress reactivity in response to a standardized psychosocial stressor. To assess stress reactivity in children, it is important to choose a stress paradigm that elicits cortisol increases (Gunnar et al., 2009). The following factors have been identified as potent psychological triggers of the HPA axis: uncontrollability, unpredictability, and social evaluation (Dickerson and Kemeny, 2004). A standardized psychosocial stress paradigm that fulfills these criteria is the well evaluated TSST-C (Buske-Kirschbaum et al., 1997).

However, the present study also has several limitations. First, we used a cross-sectional study design. Therefore, it is not possible to assess the extent to which the postnatal environment and postnatal development influence the effects of prenatal stress in later life. From animal studies (Meaney et al., 1985, 1991; Weinstock, 2008), as well as a human study (Bergman et al., 2008), it is well known that adverse effects of prenatal stress are reversible through positive postnatal maternal behavior. The offspring of mothers who received more grooming as pups, showed reduced corticosterone responses to acute stress (Liu et al., 1997). Moreover, a recent study from Bergman et al. (2010) provide first direct human evidence that the impact of increased cortisol *in utero* on impaired cognitive development, is dependent on the quality of the mother-infant relationship. Second, our three groups differed significantly in weeks of gestation as well as in length and weight at birth. It is well-established that prenatal stress is associated with abbreviated gestational length (Weinstock, 2001, 2008) and lower birth weight (French et al., 1999). The children of the TOC group had lower birth weights and shorter gestational lengths as compared to the children of the CONTROL group, but similar to the GC + TOC group. Moreover, no differences were found in cortisol levels and heart rate in reaction to the TSST-C between former preterm children and children born full-term (Buske-Kirschbaum et al., 2007). Furthermore, we ruled out the influence of the variable of whether or not parents were living together with covariate analyses. Third, it would have been helpful to measure the basal and stimulated HPA axis activity in the same study population in order to interpret our findings further. Fourth, we used a retrospective study design and the glucocorticoid administration was depended on the decisions of the responsible physicians at that time. Fifth, in the medical records was little information about the type of tocolytics or at what gestational age glucocorticoids were administered. Therefore, we couldn’t use this information in our analysis. Sixth, we did not find significant differences in salivary cortisol between the GC + TOC group and the TOC group. This may be a type II error, with the small sample not allowing for detection of a real difference.

In summary, our findings suggest that prenatal glucocorticoid exposure is associated with altered psychological and biological stress reactivity in response to a psychosocial stress test even in healthy 10-year-old children. In particular, we were able to show effects of prenatal stress on stress appraisal and recreation. Our findings suggest that prenatal stress may have long-term effects on the HPA axis regulation in 10-year-old children in reaction to a standardized psychosocial stress test. The HPA axis dysregulation is associated with various disorders and can therefore have an impact on health. Future research should focus on the factors that modulate the effects of prenatal stress on the offspring and thus influence postnatal development. Finally, a long-term follow-up of these children after puberty and in adulthood would be essential in order to define the durability of the effects found in the present study.

## Conflict of Interest Statement

The authors declare that the research was conducted in the absence of any commercial or financial relationships that could be construed as a potential conflict of interest.
